# Synergistic anti-cancer effects of silibinin-etoposide combination against human breast carcinoma MCF-7 and MDA-MB-231 cell lines 

**DOI:** 10.22038/ijbms.2021.56341.12575

**Published:** 2021-09

**Authors:** Mahdie Koushki, Azam Khedri, Mohammad Aberomand, Kourosh Akbari Baghbani, Ghorban Mohammadzadeh

**Affiliations:** 1 Department of Clinical Biochemistry, Ahvaz Jundishapur University of Medical Sciences, Ahvaz, Iran; 2 Toxicology Research Center, Department of Clinical Biochemistry, Faculty of Medicine, Ahvaz Jundishapur University of Medical Sciences, Ahvaz, Iran; 3 Department of Infection, Immunity, and Inflammation, University of Leicester, LE1 7RH, UK; 4 Hyperlipidemia Research Center, Department of Clinical Biochemistry, Faculty of Medicine, Ahvaz Jundishapur University of Medical Sciences, Ahvaz, Iran

**Keywords:** Apoptosis, Breast cancer, Drug synergism, Etoposide, MCF-7 cells, Silibinin

## Abstract

**Objective(s):**

Recently, there is a significant focus on combination chemotherapy for cancer using a cytotoxic drug and a phytochemical compound. We investigated the effect of silibinin on etoposide-induced apoptosis in MCF-7 and MDA-MB-231 breast carcinoma cell lines.

**Materials and Methods:**

The cytotoxic effects of silibinin and etoposide were determined using MTT assay after 24 and 48 hr incubation with these drugs individually and combined. The mRNA expression of Bax and Bcl2, and protein levels of P53, phosphorylated p53 (P-P53), and P21 were determined using real-time PCR and western blot analysis, respectively. The caspase 9 activity was measured using an ELISA kit.

**Results:**

Silibinin and etoposide alone and combined significantly inhibit cell growth in a dose and time-dependent manner in both cell lines. The strongest synergistic effects in terms of MCF-7 cell growth inhibition [combination index (CI) = 0.066] were evident. The silibinin-etoposide combinations cause a much powerful apoptotic death (47% and 40%) compared with each compound individually in MCF-7 and MDA-MB 231 cells, respectively. Additionally, the silibinin-etoposide combinations significantly increased the expression of P53, P-P53, and P21 in MCF-7 cells. Neither silibinin nor etoposide individually increased the level of P53 and P-P53 in MDA-MB-231 cells, but both of them individually and combined increased the level of P21.

**Conclusion:**

Since the silibinin-etoposide combination induces apoptosis in both cell lines with and without expression of p53, thus, it is suggested that this combination may be a successful therapeutic strategy for breast cancer regardless of P53 status.

## Introduction

Breast cancer, a multifactorial and highly heterogeneous disease, is one of the most important causes of mortality in women worldwide (1). While, recently, various systemic therapies such as chemotherapy, hormone therapy, and immunotherapy are used for treatment of breast cancer, chemotherapy is still the most common type of therapy, but in terms of growth inhibition, metastasis, and recurrence it is ineffective. (2). Conventional chemotherapy is one of the main challenges for breast cancer due to several undesirable side effects on the non-cancerous cells and development of multidrug resistance (3). Therefore, reduction of dose-limited side effects and increasing the efficacy of the desired drug is one of the most important challenges in conventional chemotherapy (4-6). One of the relatively new and valuable therapies against cancer is combination chemotherapy, using nontoxic or very low toxicity phytochemical compounds combined with common chemotherapeutic agents, for reducing their cytotoxicity effect on non-cancerous cells and synergistically increasing their anticancer effects (7). Therefore, various phytochemical compounds have been studied due to their anticancer effects as potential candidates for combination chemotherapy (8). The anticancer influence of flavonoids on various cancer cell lines and their synergistic effects in some cases have been reported recently (9). 

Silibinin, the most abundant compound in silymarin, has anti-oxidant (10, 11), anticancer (12, 13), and apoptotic effects, and in patients with prostate cancer it is in phases I and II of clinical trial (14-17). The silibinin apoptotic effects on breast cancer cell lines have been demonstrated previously. (14). Additionally, recently, the synergistic effects of silibinin combined with several chemotherapeutic drugs have been reported in a review article (18). 

Etoposide, a potent topoisomerase II inhibitor, is an antineoplastic and widely used drug for malignancy chemotherapy that couples DNA damage to cellular apoptosis (19). Cellular responses to DNA damage include cell cycle arrest and DNA repair, but ultimately cell death occurs if repair is unsuccessful. Etoposide accumulates cells at the G2/M phase, which can occur in both p53-dependent and independent pathways (20). The cell cycle blockage stimulated by DNA damage is regulated by DNA-dependent protein kinases in the p53-independent pathway. On the other hand, the p53-dependent pathway succeeds the arrest at the G2 phase by p53 mediated suppression of cyclin B/ CDK promoters (21). Although previous studies demonstrated the anticancer effects of silibinin and etoposide individually, the silibinin-etoposide combination may have a potent anticancer effect. According to our knowledge, this combination has not been examined, consequently, we investigated the anticancer effects of the silibinin-etoposide combination on the MCF-and MDA-MB-231 of breast carcinoma cell lines.

## Materials and Methods

***Determination of cell growth and combination index (CI) calculation***

MCF-7 and MDA-MB-231 human breast carcinoma cell lines were purchased from the National Cell Bank, Pasteur Institute of Iran (Tehran, Iran). Briefly, 3 × 10^3^ cells per well were seeded into 96-well plates and various doses of silibinin and etoposide individually and combined were treated for 24 and 48 hr. Next, 20 μl MTT solution (5 mg/ml) was added to each well and incubated for 4 hr at 37 °C. Finally, after removing the culture medium, in order to dissolve formazan crystals, pure DMSO (200 μl per well) was added. The absorbance of the wells was measured using a microplate reader (BioTek® ELx800, USA) at 570 nm. The concentration of a drug with 50% cell viability inhibition was considered as the half-maximal inhibitory concentration (IC_50_) value. The combination index (CI) between silibinin and etoposide was calculated using CompuSyn software according to Chou *et al*. equation (22). By definition, CI < 1.0 is described as synergism, CI > 1.0 as antagonism, and CI = 1.0 as additive.

***Analysis of apoptosis by Annexin/PI-based flow cytometry***

The apoptotic effect of the silibinin-etoposide combination was measured using a commercially available kit (IQ Products, Groningen, Netherlands). Briefly, 5×10^5^ cells were plated and incubated overnight for attachment, and then treated with different concentrations of silibinin (5–100 μM) combined with 5 μM of etoposide for 24 hr. After trypsinization and twice washing with cold phosphate buffer saline (PBS), the cells were re-suspended in annexin V binding buffer (1x). Lastly, after staining the cells with annexin V/PI at room temperature for 15 min the cells were counted using a Becton Dickinson flow cytometer. The quadrant analysis was used to measure the percentage of apoptotic cells based on early and late apoptotic areas in comparison with the total cell population.

***Determination of Bax and Bcl***_2_ ***expression, and caspase activity using qRT-PCR and ELISA kit***

After isolation of total RNA from the cells using a commercially available kit (Qiagen, Germany), the quantity and quality of extracted RNA were determined with a spectrophotometer (NanoDrop 2000, Thermo Fisher Scientific, USA) and 1.0% agarose gel electrophoresis, respectively. For c.DNA synthesis 1 μg of total RNA was subjected to reverse transcription-PCR using a commercially available kit (Thermo Fisher Scientific). cDNA amplification was performed using the following primer sets: Bax (5’-GGGTGGTTGGGTGAGACTC-3’, 5’-AGACACGT AAGGAAAACGCATTA-3’); Bcl2(5′-TCGCCCTGTGGATGA CTGA-3′,5′CAGAGACAGCCAG GAGAAATCA-3′); and β-Actin (5’-TGGACTTCGAGCAAGAGATG-3’, 5’-GAAGGAA

GGCTGGAAGAGTG-3’). The comparative Ct method (ΔΔCt method) was used to determine the relative expression of the target genes in comparison with the β-actin housekeeping gene. For measurement of caspase 9 activity, after obtaining the cell lysate in ice-cold RIPA buffer, the enzyme activity was determined using a commercially available ELISA kit.

***Determination of p53, phosphorylated-p53, and P21 expression using western blotting***

Approximately 3×10^5^ cells per well were seeded and overnight incubated with different concentrations of silibinin (5, 10, 25, 50, and 100 μM) and etoposide (5,10, 25, 50, and 100 μM) individually, and 5,10, 25, 50, and 100 μM of silibinin combined with 5 μM of etoposide at 37 °C. Proteins were extracted from the cells using ice-cold radioimmunoprecipitation buffer (RIPA) supplemented with the proteases/phosphatase cocktail inhibitor. The total protein concentration of the lysate was determined using a bicinchoninic acid (BCA) kit (Parstous Biotechnology, Iran). A total of 50 μg proteins from each sample were run on 12% sodium dodecyl sulfate-polyacrylamide electrophoresis (SDS-PAGE) and electrotransferred to polyvinylidene difluoride (PVDF) membranes (Millipore, Bedford, MA, USA). After blocking the membranes for 4 hr at room temperature, they were incubated with specific antibodies against P53 (9282s) Phospho-P53 (ser 15, sc-20150), p21 (sc-20150), and β-actin (sc-130656) overnight at 4 °C and then incubated with HRP-conjugated secondary antibody for 1 hr. The blots were then finally detected using an ECL (enhanced chemiluminescence) detection kit and quantified by densitometry using a ChemiDocTM system (Bio-Rad, Hercules, CA, USA).

***Statistical analysis***

All quantitative data are expressed as mean ± SEM of three independent experiments. The Student’s t-test was used to determine the difference between the two groups. One-way ANOVA was conducted for multiple-group comparison statistical analysis by using the Prism statistical software package (GraphPad Software, USA). Statistical significance was accepted at the level of *P*<0.05.

## Results

***The silibinin-etoposide combination showed a synergistic effect***

**Figure 1:**
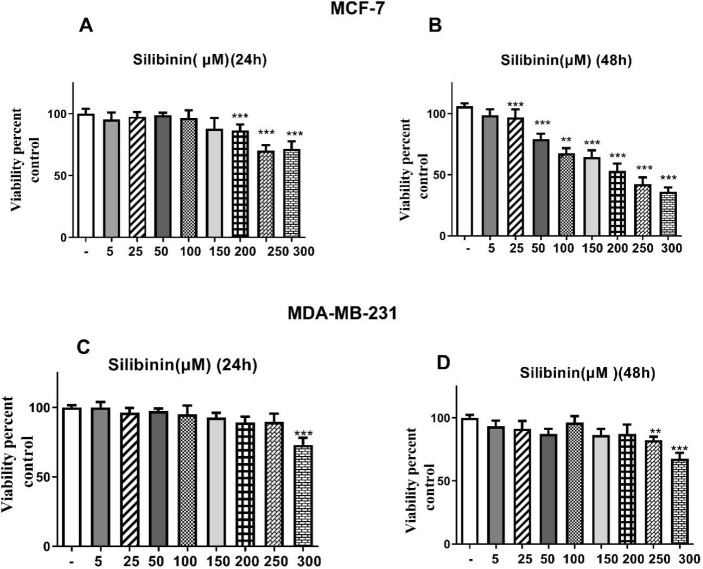
Cytotoxic effects of silibinin on the growth of both cell lines. The cells in DMEM medium containing 10% FBS were treated with various concentrations of silibinin (5-300 µM) for 24 hr (A, C) and 48 hr (B, D) and then subjected to MTT assay. The results are presented as mean ± standard error of the mean (SEM) for triplicates. (*P<0.05, **P<0.01, ***P<0.001 vs untreated control)

**Figure 2:**
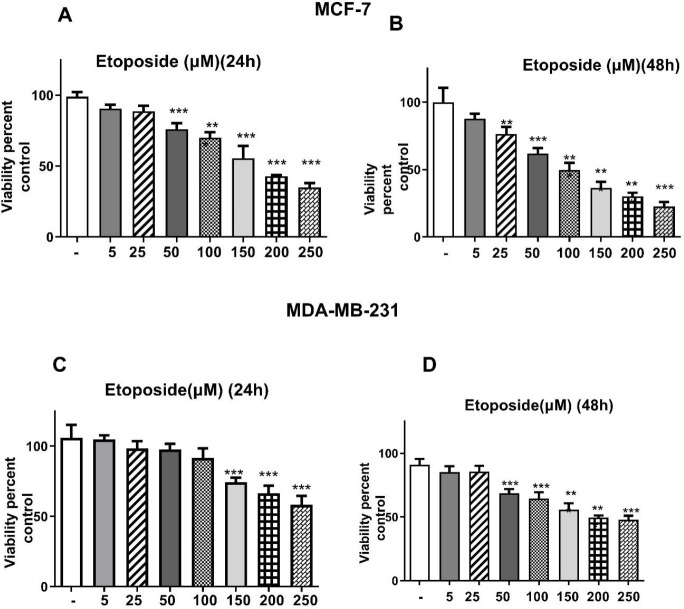
Cytotoxic effects of etoposide on the growth of both cell lines. The cells in DMEM medium containing 10% FBS were treated with various concentrations of etoposide (5–250 µM) for 24 hr (A, C) and 48 hr (B, D) and then subjected to MTT assay. The results are presented as mean ± SEM for triplicates. (*P<0.05, **P<0.01, ***P<0.001 vs untreated control)

**Figure 3:**
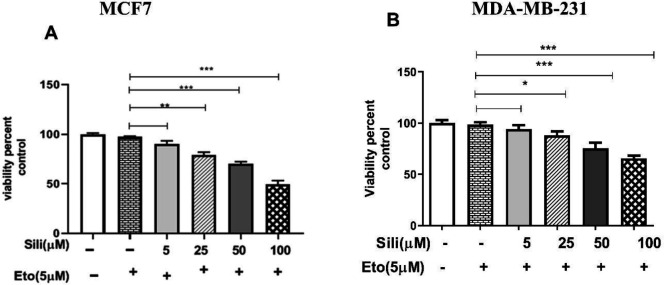
Synergistic cytotoxic effect of the silibinin-etoposide combination on the growth of both cell lines. The cells in DMEM medium containing 10% FBS were treated with indicated concentrations of the silibinin-etoposide combination for 24 hr then subjected to MTT assay. The results are presented as mean ± SEM for triplicates. (*P<0.05, **P<0.01, ***P<0.001 vs untreated control)

Our results indicated that silibinin shows a toxic effect on cell viability and its least toxic effect was observed after 48 hr of incubation with 25 and 250 µM in MCF-7 and MDA-MB-231 cells, respectively (Figure 1). After 48 hr of incubation with silibinin, the IC_50_ value was calculated with 200 μM in MCF-7 cells. Additionally, etoposide showed a toxic effect on the cell viability, and its least toxic effect was observed after 48 hr of incubation with 25 and 150 µM in MCF-7 and MDA-MB-231 cells, respectively (Figure 2). The IC_50_ value was estimated to be about 150 μM in MCF-7 cells after 24 hr incubation with etoposide individually. Moreover, the IC_50_ value was reduced to 100 and 200 μM in MCF-7 and MDA-MB-231 cells, respectively, after treatment for 48 hr (Figure 2). As shown in Figure 3, all silibinin-etoposide combinations had a greater toxic effect on cell viability in both cell lines. A combination of 100 μM of silibinin with 5 μM of etoposide for 24 hr caused 50% and 30% inhibition in MCF-7, and MDA-MB-231 cells, respectively. Next, by using CompuSyn software the possibility of a synergistic effect between silibinin and etoposide was investigated. As shown in Table 1, all combinations between silibinin and etoposide had a synergistic effect on cell viability of MCF-7. The most synergistic effect (CI= 0.066), was observed at silibinin 100 μM plus etoposide 10 μM (Table 1).

***The silibinin-etoposide combination showed a synergistic effect on the mRNA expression of apoptosis markers***

After staining with Annexin V/PI, our results indicated that the silibinin-etoposide combination could induce apoptotic death dose-dependently in both cell lines. The total apoptotic population significantly increased in MCF-7 (58%) and MDA-MB-231 (38%) treated with a combination of 5 μM etoposide and 100 μM silibinin for 24 hr. These findings indicated that the silibinin-etoposide combination could significantly increase the apoptotic death in both cell lines in comparison with etoposide individually (*P*<0.001). Additionally, our findings showed that the expression of Bax was significantly increased in MCF-7 and MDA-MB-231 (12.5-fold and 7.5-fold, respectively) with silibinin 100 μM plus etoposide 5 μM (Figures 5A and B). While the expression of Bcl_2_ was significantly decreased in MCF-7 and MDA-MB-231 (2.0-fold and 10.0-fold, respectively) (Figures 5C and D). Furthermore, the results observed from apoptosis analysis were consistent with the results obtained from the mRNA expression of the apoptotic biomarkers. On the other hand, our results showed that the silibinin-etoposide combination could increase caspase 9 activity dose-dependently in both cell lines (Figure 6). Consequently, the combination-induced apoptotic death may be dependent on caspase activation.

***The silibinin-etoposide combination showed a synergistic effect on P53, P-P53, and P21 expression***

Our results showed that after 24 hr of treatment with etoposide individually the protein levels of p53, P-P53, and P21 were significantly increased dose-dependently in MCF-7 cells (Figure 7). Similarly, after treatment of MCF-7 cells with silibinin individually the protein level of p53, P-P53, and P21 were significantly increased dose-dependently (Figure 7). Additionally, after treatment of MCF-7 cells with silibinin-etoposide combination, the protein levels of p53, P-P53, and P21 were significantly increased dose-dependently (Figure 8). Therefore, we demonstrated that induction of protein P21 expression by silibinin and etoposide either individually or combined might be dependent on P53 activation in MCF-7 cells. Whereas, our results in MDA-MB-231 cells indicated that silibinin and etoposide neither individually nor combined affect the expression of p53 and P-P53, whereas the expression of p21 was significantly increased by silibinin and etoposide either individually or combined (Figures 9 and 10). Therefore, we found induction of P21 expression in MDA-MB-231 cells by silibinin and etoposide either individually or combined may be independent of p53 activation.

**Table 1:** Combination Index (CI) between silibinin and etoposide in MCF-7 cells

Silibinin (100 µM)	Silibinin (50 µM)	Silibinin (25 µM)	Silibinin (5 µM)	
0.1	0.38	0.59	0.86	Etoposide (5 µM)
0.06	0.1	0.2	0.96	Etoposide (10 µM)

CI < 1.0 represents synergism, CI = 1.0 represents an additive, and CI >1.0 represents antagonism

**Figure 4:**
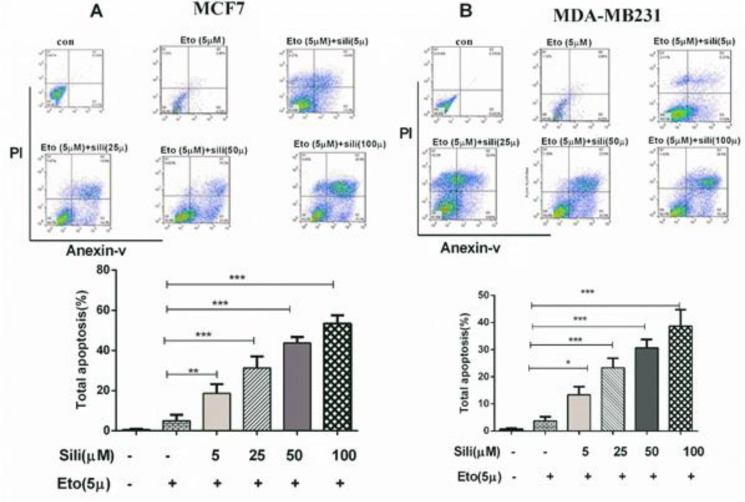
Flow cytometric analysis of the combination-induced apoptotic death in both cell lines. The cells were treated with various concentrations of silibinin (5–100 μM) combined with 5 μM of etoposide for 24 hr. Apoptosis bar graphs (b) represent mean ± SEM, n= 3. Differences between means and significance of the treatments were analyzed using ANOVA and Tukey’s multiple comparison test. *P<0.05 vs control, **P<0.01 vs control, and ***P<0.001 vs control

**Figure 5:**
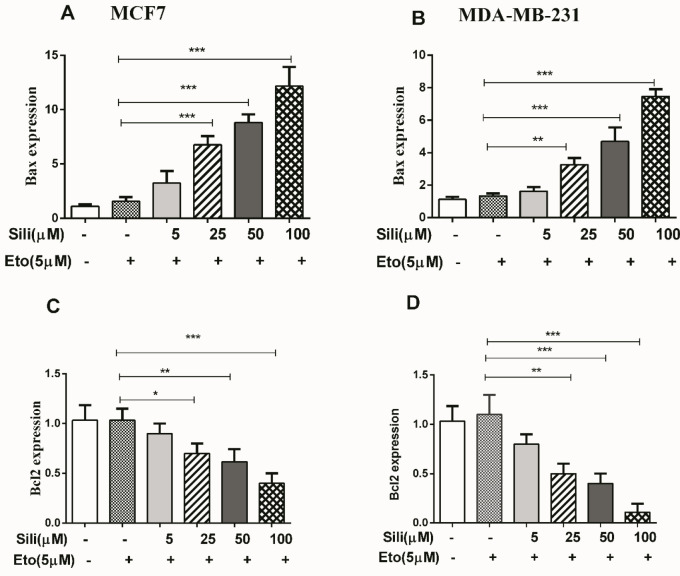
The mRNA expression of Bax, Bcl2, and P21 after treatment with the silibinin-etoposide combination in both cell lines. The cells were treated with various concentrations of silibinin (5–100 μM) combined with 5 μM of etoposide for 24 hr. The data are presented as means ± SEM of three experiments. Statistical significant was accepted if *P*<0.05. *P<0.05, **P<0.01, ***P<0.001 vs untreated control

**Figure 6:**
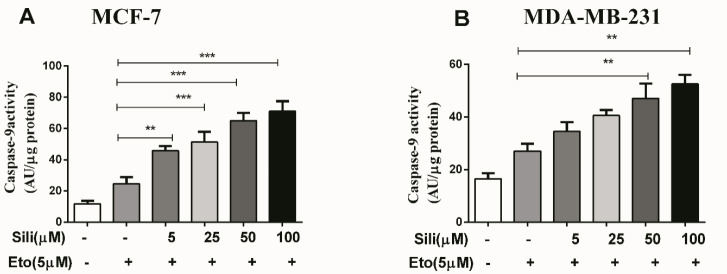
The silibinin-etoposide combination increased caspase 9 activity in both cell lines. The cells were treated with different concentrations of silibinin (5–100 μM) combined with 5 μM of etoposide for 24 hr. The data are presented as means ± SEM of three experiments. Statistical significant was accepted if *P<0.05. *P<0.05, **P<0.01, ***P<0.001 vs untreated control

**Figure 7:**
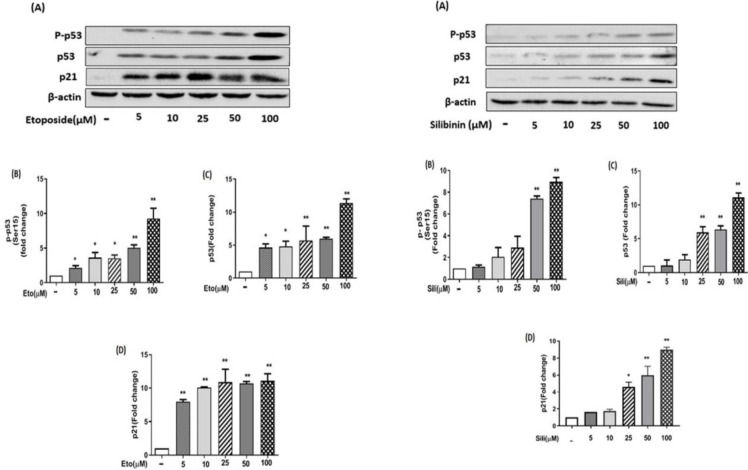
Etoposide and silibinin individually increased the expression of p53, P-P53, and p21 in MCF-7 cells. The cells were treated with different concentrations (5-100 μM) of silibinin for 24 hr. The data are presented as the mean ± SEM of three experiments. Fold changes were calculated relative to untreated groups using an independent-samples t-test. *P<0.05, **P<0.01, ***P<0.001 vs untreated control

**Figure 8:**
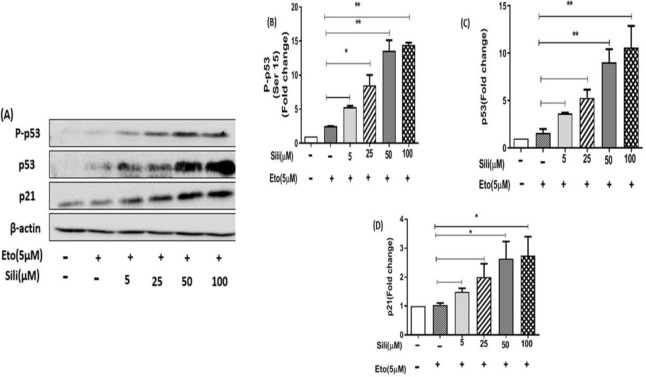
Silibinin-etoposide combination increased the expression of p53, P-P53, and p21 in MCF-7 cells. The cells were treated with various concentrations of silibinin (5–100 μM) combined with 5 μM of etoposide for 24 hr. The data are presented as mean ± SEM of three experiments. Fold changes were calculated relative to untreated groups using an independent-samples t-test. *P<0.05, **P<0.01, ***P<0.001 vs untreated control

**Figure 9:**
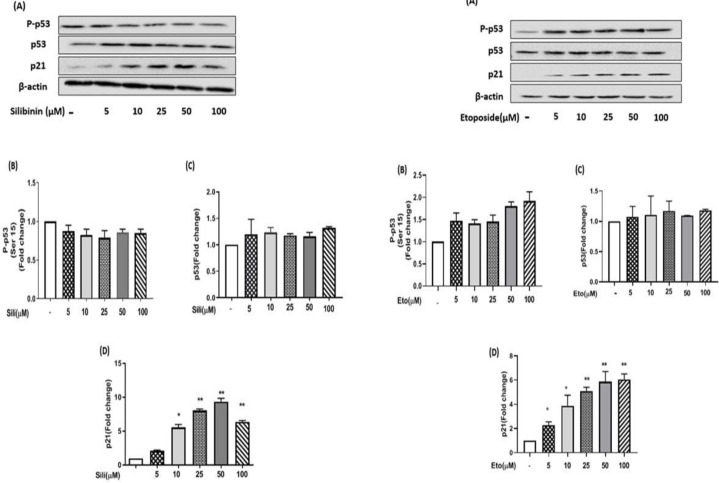
Silibinin and etoposide individually could not increase the expression of P53, P-P53, and P21 in MDA-MB-231 cells. The cells were treated with different concentrations of silibinin (5–100 μM) and etoposide (5–100 μM) individually for 24 hr in the normal medium. The data are presented as the mean ± SEM of three experiments. Fold changes were calculated relative to untreated groups using an independent-samples t-test. *P<0.05, **P<0.01, ***P<0.001 vs untreated control

**Figure 10:**
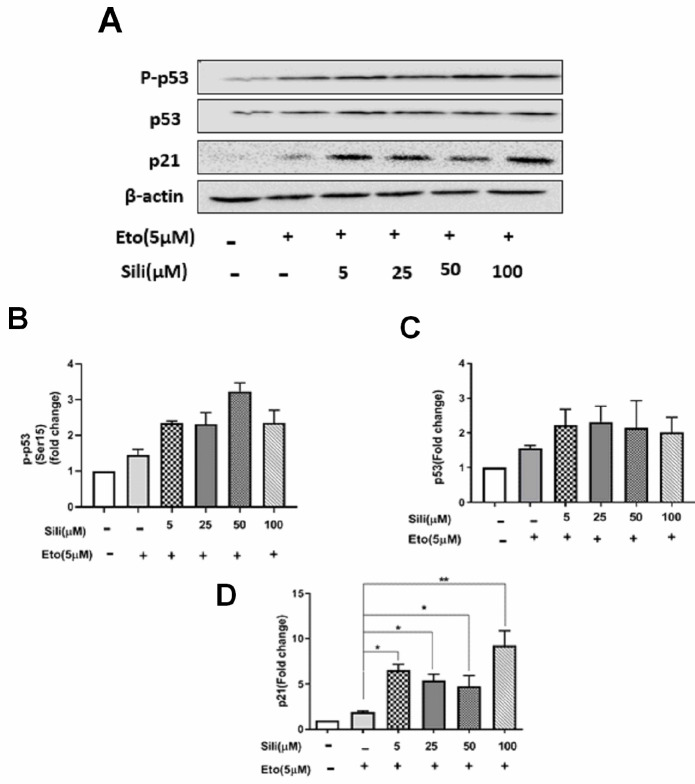
Silibinin-etoposide combination could not increase P53 or P-P53 expression but could increase P21 expression in MDA-MB-231 cells. The cells were treated with various concentrations of silibinin (5–100 μM) combined with 5 μM of etoposide for 24 hr. The data are presented as the mean ± SEM of three experiments. Fold changes were calculated relative to untreated groups using an independent-samples t-test. *P<0.05, **P<0.01 vs untreated control

## Discussion

The main result of our study is that silibinin could significantly increase the etoposide efficacy in terms of anticancer effects on the MCF-7 and MDA-MB-231 cells. This result suggests that the appropriate combination of etoposide with silibinin, which was previously used clinically, may have a potential beneficial effect on breast cancer. This finding is mainly important, since, etoposide and other cytotoxic anticancer drugs, have several undesirable side effects on the non-cancerous cells (23). Moreover, the clinical benefit of these drugs in cancerous cells will be restricted due to heritable and acquired resistance (6, 24, 25). Based on this idea, our results indicated that combination chemotherapy may have a clinically beneficial effect on cancer patients rather than single chemotherapy. On the other hand, a natural compound with a potent anti-tumor effect has the potential to be considered as an agent in combination chemotherapy, which may reduce the side effects of chemotherapeutic agents (4, 8, 26).

Generally, we showed 100 μM silibinin combined with 5 μM etoposide could significantly decrease the therapeutic concentration of etoposide at nearly one-thirtieth in both cell lines. Additionally, we revealed the therapeutic effect of etoposide combined with silibinin was significantly increased in both breast carcinoma cell lines. These results strongly support the theory that a combination of a phytochemical agent such as silibinin with a cytotoxic agent might maintain its benefitial anticancer effects but minimize its undesirable side effects. Our results showed that co-treatment of both cell lines with 100 μM silibinin plus 5 μM etoposide could significantly inhibit cell viability. Silibinin individually or combined with etoposide could increase the level of P53 expression that causes a significant increase in the level of P21 expression in MCF-7 cells. Moreover, silibinin individually or combined with etoposide could increase the phosphorylation of P53 in MCF-7 cells, which is widely used as an indicator of cell apoptosis (27). Additionally, the silibinin-etoposide combination could increase the level of P21 expression in MDA-MB-223 cells. Consequently, our results suggest that the silibinin-etoposide combination more effectively induced apoptotic death in comparison with both of them individually in both cell lines and at a dose that possibly is not toxic to normal cells. Previous studies indicated that P53 has an important role in carcinogenesis and cancer progression (28- 30). In a study conducted by Agarwal *et al*., they showed that silibinin causes cell-cycle arrest at the G1 phase in HT-29 human colon carcinoma cells. (31). Additionally, the inhibition of growth with silibinin in human colorectal carcinoma cells (HCT-116) has been reported (32). On the other hand, the inhibition of invasion, motility, and migration in a gastric cell line with silibinin through down-regulation of MMP-2 and MMP-9 expression has been reported by another study (33). For the first time, we showed that the combination of 100 μM silibinin plus 5 μM etoposide could more efficiently induce apoptosis than treatment with 5 μM etoposide individually in both cell lines. It has been demonstrated that apoptosis has a key role in the development and progression of several solid tumors and it has the potential to be considered for targeting therapy in several tumors such as breast cancer (34). Based on this fact, because the most anti carcinogenic mechanism of these drugs in breast cancer cells is induction of apoptosis, thus, our results are important. Furthermore, the increased level of P-P53 and P21 in both cell lines demonstrated that apoptosis is the predominant mechanism of the silibinin-etoposide combination anticancer effect. More studies are required in order to determine whether other silibinin-etoposide combinations that show synergistic effects can enhance apoptotic death. Furthermore, other studies are also needed to determine the molecular mechanism of the combination-induced apoptosis.

Resistance or escape of apoptotic death is the main mechanism for drug resistance, which causes the development and progression of several solid tumors (34). Both intrinsic and extrinsic pathways are involved in the activation of apoptosis. It has been demonstrated drug therapy-induced apoptosis can increase cytochrome C releasing from mitochondria by induction of p53 expression. Then, cytochrome C-induced activated caspase 9 can activate caspase 3 (34). Etoposide (35) and silibinin (36) have been shown to induce cancer cell apoptotic death through both intrinsic and extrinsic pathways. In the current study, first, the effect of silibinin and etoposide individually and combined on the levels of P53, P-P53, and P21 were investigated in both cell lines. Then, we studied the effect of silibinin and etoposide individually and combined on the typical downstream markers of P53 including Bax as a pro-apoptotic and Bcl_2_ as an anti-apoptotic, and P21 as a cell cycle inhibitory protein. Our findings indicated that silibinin and etoposide individually and combined could significantly and dose-dependently increase the protein levels of P53, P-P53, and P21 (maybe their expression is exactly associated with p53) in MCF-7 cells at 24 hr. Moreover, a significant and dose-dependent increase in mRNA expression of Bax and caspase 9 activity was observed at 24 hr. These results are consistent with the results of a another study (32). Whereas, neither silibinin nor etoposide individually or combined could increase the protein levels of p53 and P-P53 expression in MDA-MB-231 cells at the same time. However, the protein level of P21 in MDA-MB-231 cells was significantly increased after treatment with both of them individually and combined, maybe its expression is exactly independent of P53.

In the current study, the percentage of apoptotic and necrotic death in treated cells with silibinin and etoposide individually and combined was determined by flow cytometry analysis. The results indicated that silibinin individually and combined with etoposide could increase the percentage of apoptotic death in both cell lines. Combined treatment with silibinin and a nontoxic dose of etoposide showed a synergistic effect between the two drugs, which causes a 3-fold increase in apoptotic death. Etoposide, a potent topoisomerase II inhibitor (37), causes arrest in the cells at the gap II/mitosis phase due to its inhibitory effect on the progression of the synthesis phase of the cell cycle (38). In addition to G2/M arrest, which causes cell cycle-dependent death, etoposide could induce apoptosis through mitochondrial-dependent action of p53(39). On the other hand, the effects of polyphenols on the anticancer activity of etoposide in different conditions have been reported in a review article (38). The association between tumor suppressor function, modulation of apoptosis, and inhibition of tumor cell proliferation is controversial; although, some studies have shown various cancer cell lines with overexpression of p53 are more sensitive to apoptotic death due to its direct involvement in the initiation of apoptosis (40). Another study reported that silibinin could inhibit cell proliferation and increase apoptosis independent of P53 status (41).

## Conclusion

We demonstrated that silibinin synergistically inhibits the growth of MCF-7 and MDA-MB-231 cell lines combined with etoposide and sensitizes both of them to the toxic effects of this clinically used chemotherapeutic agent. The stimulatory effects of silibinin on the expression of some key apoptosis-associated proteins including P53, P-P53, and P21 were related to growth inhibition and enhanced the apoptotic death of etoposide in both cell lines. Our results confirmed the previous studies indicated that silibinin sensitizes cancer cells to anticancer drugs and supply credible proof for the therapeutic potential of this natural compound for treating some drug-insensitive breast cancers. Taken together, since the silibinin-etoposide combination induces apoptosis in both cell lines with and without expression of p53, thus, it is suggested that this combination may be a successful therapeutic strategy for breast cancer regardless of P53 status.

## Authors’ Contributions

GM, AK, and MA contributed to the conception and design of the study. AK carried out the flow cytometry and Western blot analysis, MK and KAB carried out all the other experiments and contributed to the interpretation of the results. GM drafted the manuscript and final revised the manuscript critically for publication in the journal. The corresponding author declares that all listed authors meet the authorship criteria and that no other authors meeting the criteria have been omitted.

## Conflicts of Interest

The authors declare no conflicts of interest. The authors are fully responsible for data collection, originality, interpretation, and writing of this manuscript.
